# A Low-Cost, Multi-Sensor System to Monitor Temporary Stream Dynamics in Mountainous Headwater Catchments

**DOI:** 10.3390/s19214645

**Published:** 2019-10-25

**Authors:** Rick S. Assendelft, H.J. Ilja van Meerveld

**Affiliations:** Department of Geography, University of Zurich, Winterthurerstrasse 190, 8057 Zürich, Switzerland; ilja.vanmeerveld@geo.uzh.ch

**Keywords:** intermittent streams, ephemeral streams, monitoring, hydrological state, Arduino, low-cost sensors, DIY sensors, time-lapse cameras, sensor evaluation, stream network

## Abstract

While temporary streams account for more than half of the global discharge, high spatiotemporal resolution data on the three main hydrological states (dry streambed, standing water, and flowing water) of temporary stream remains sparse. This study presents a low-cost, multi-sensor system to monitor the hydrological state of temporary streams in mountainous headwaters. The monitoring system consists of an Arduino microcontroller board combined with an SD-card data logger shield, and four sensors: an electrical resistance (ER) sensor, temperature sensor, float switch sensor, and flow sensor. The monitoring system was tested in a small mountainous headwater catchment, where it was installed on multiple locations in the stream network, during two field seasons (2016 and 2017). Time-lapse cameras were installed at all monitoring system locations to evaluate the sensor performance. The field tests showed that the monitoring system was power efficient (running for nine months on four AA batteries at a five-minute logging interval) and able to reliably log data (<1% failed data logs). Of the sensors, the ER sensor (99.9% correct state data and 90.9% correctly timed state changes) and flow sensor (99.9% correct state data and 90.5% correctly timed state changes) performed best (2017 performance results). A setup of the monitoring system with these sensors can provide long-term, high spatiotemporal resolution data on the hydrological state of temporary streams, which will help to improve our understanding of the hydrological functioning of these important systems.

## 1. Introduction

There are three main hydrological states for temporary streams: dry streambed, standing water, and flowing water [1,2]. Temporary streams alternate between at least two of these states as a result of seasonal changes in catchment wetness, and in direct response to rainfall and snowmelt events [3]. Temporary streams are valuable ecosystems at the transition between aquatic and terrestrial environments. While they are most common in arid and semi-arid regions, temporary streams are found in all climatic zones around the world [4,5], often in the headwaters of perennial streams [6,7]. Estimates suggest that their total length and discharge account for at least half of the global stream network [8]. Because climate change, water abstraction, and land-use change alter the flow regimes of perennial streams, the number of temporary streams is expected to increase in the near future [4,9].

The recognition of the ubiquity of temporary streams and the concern over their vulnerability to climate change and other human disturbances have led to an increased number of studies on these systems in the last decades. The majority of these studies have focused on the ecological and biochemical functioning of temporary streams [8,10] and have highlighted their importance as: unique animal and plant habitats with high biodiversity [5,11]; migration corridors [12,13]; sources and sinks of organic matter and nutrients [4]; and biochemical hotspots with high reaction rates compared to neighboring environments [14]. 

In comparison, there are fewer hydrological studies on temporary streams. To improve models, policy and conservation practices, scientists have expressed the need to better understand the hydrological functioning of temporary streams [13,15,16,17,18]. A large part of the available hydrological research on temporary streams has focused on their role as sources of groundwater recharge, specifically in arid regions [19,20,21,22,23]. Relatively less studied are the spatiotemporal dynamics in stream network extension and connection as a result of hydrological state changes in temporary streams. After research in the 1960s–1970s [24,25,26,27,28] could not establish an explicit relationship between drainage density and hydrological response, few other studies on stream network dynamics were conducted in the following decades [29]. However, recent studies have shown the importance of obtaining insight into these dynamics as they reflect (sub)surface storage patterns and streamflow generation processes [29,30], and could have a significant effect on downstream (perennial) discharge [31,32] and water quality [33,34]. 

It is therefore essential to collect high spatiotemporal resolution data on the hydrological state of temporary streams. However, because monitoring temporary streams is difficult due to their flashy and erosive nature, often high sediment loads, and limited accessibility [3,23], this kind of data remains sparse. Conventional methods to monitor perennial streams, including stream gauges, current meters, and pressure transducers, are generally less practical and cost-effective for high spatiotemporal resolution monitoring of the hydrological state of temporary streams [35]. Several studies have monitored the changes in the hydrological state of temporary streams by mapping the extent of the streams through direct observation. This is laborious and logistically challenging [29], especially during rainfall events when conditions change quickly [26,28]. Most of these mapping studies are therefore limited to describing seasonal changes in stream network dynamics [29,36,37,38]. Other mapping methods include aerial photography [39,40], LiDAR data [41], and unmanned aerial systems [42]. Aerial photography and LiDAR data can provide information for large areas, but, due to high costs, are unsuitable for continuous monitoring. Furthermore, they can only be used in catchments where the wet channel is exposed and of a certain dimension. This excludes forested catchments and small headwater streams. Unmanned aerial systems have the potential to be more cost-effective and provide higher resolution data but are equally ill-suited for temporary stream monitoring in densely vegetated catchments. In addition, operation might be problematic during intense rainfall events. 

A growing number of hydrologists have addressed the challenges and limitations of traditional hydrological monitoring approaches by developing or modifying low-cost sensors [43]. Several studies aimed at collecting information on the hydrological state of temporary streams have used the same approach. Temperature sensors have been used to obtain information about the presence of water in temporary streams by looking at the diurnal temperature signal of the streambed, which has a larger amplitude in case of a dry streambed than in case of a wet streambed [44,45]. The downside of this approach is the complexity and subjectivity of the data analysis. It can be especially difficult to identify instances when water is present for a short period (i.e., for a few hours during events) or to distinguish between the onset of channel wetting and sudden weather-related shifts in temperature. Electrical resistance (ER) sensors [35,46,47,48] and float switch sensors [49] have also been used to determine the presence of water in temporary streams. ER sensors measure the resistance between two electrodes, which is relatively low when water is present and high when water is absent. Float switch sensors consist of a float with a magnet, and a reed switch, and detect the presence of water when the water level rises and aligns the float with the reed switch, causing the magnet to open or close the switch (depending on the type of reed switch). In comparison to temperature sensors, ER and float switch sensors generally provide more accurate and easily interpretable data, while the costs and the potential for high spatiotemporal resolution data are similar. However, the major shortcoming of these three sensors is their inability to discriminate between the standing water and flowing water states. Bhamjee et al. (2016) [50], addressed this problem by pairing an ER sensor with a custom-made vane flow sensor. While the design of their flow sensor was not without limitations (particularly its susceptibility to sediment related issues), their study showed the added value of a flow sensor for comprehensive monitoring of the hydrological state of temporary streams.

Despite the increase in the number of studies using low-cost sensor approaches to collect high spatiotemporal resolution data on the hydrological state of temporary streams, there is, with the exception of Bhamjee et al. (2016) [50], a clear lack of low-cost approaches that provide information on all three of the main hydrological states. Furthermore, previous studies were mainly conducted in rural [46], agricultural [35,50], and peatland catchments [48]. Comparable low-cost sensor approaches in mountainous headwater catchments where hydrological conditions can be more dynamic and sediment loads larger, have yet to be tested. Finally, the field performance of the low-cost sensors from earlier research was primarily evaluated by assessing the robustness of the sensor [35,44,48,50], determining the amount of noise in the sensor data [35,50], comparing the sensor data with upstream hydrometric data [44], or analysing the validity of the combined output of two sensors [50]. Sensor evaluation based on actual comparison of the sensor data with continuous direct observations, which provides a more comprehensive insight into sensor performance, has so far been limited.

In order to fill these gaps, this study takes advantage of the recent rise of inexpensive, open-source technology and resources [51] by using microcontroller boards, low-cost modules and sensors, a 3D printer, and time-lapse cameras to create, program, and comprehensively test and evaluate a versatile, low-cost, multi-sensor system tailored for monitoring the presence of water and the occurrence of flow in small temporary streams in mountainous headwater catchments.

## 2. Multi-Sensor Monitoring System

**Figure 1:**
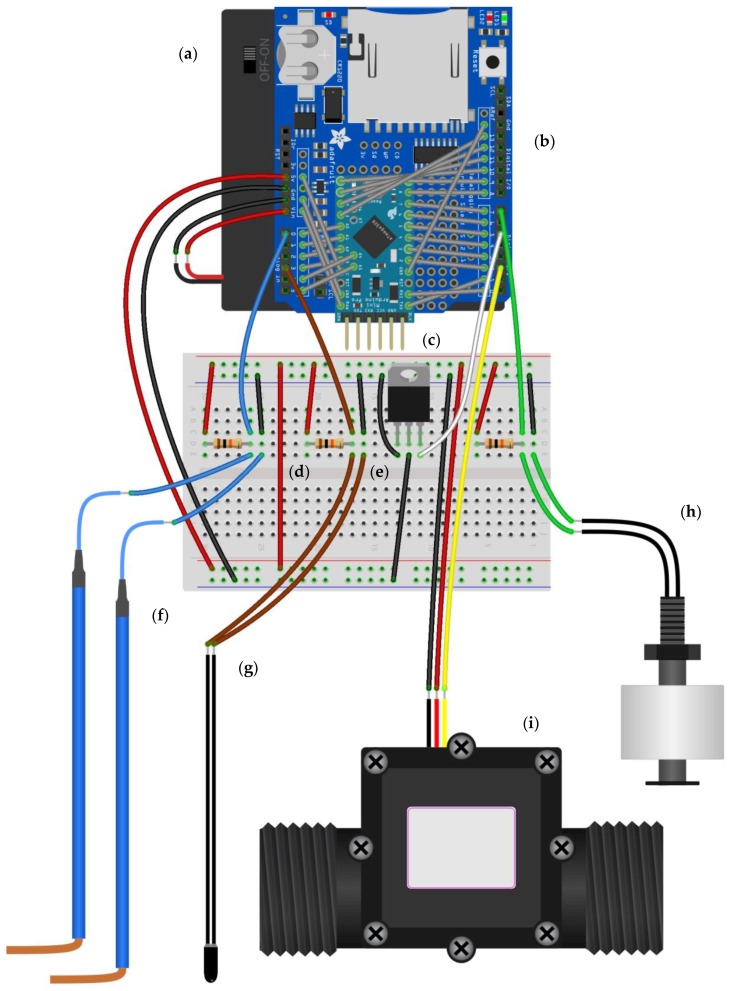
Wiring diagram of the multi-sensor monitoring system. The main components include: (**a**) battery pack for four AA batteries, (**b**) SD-card data logger shield, (**c**) Arduino Pro Mini microcontroller board with six-pin header for programming, (**d**) 10 KΩ resistors, (**e**) N-channel MOSFET, (**f**) ER sensor, (**g**) temperature sensor, (**h**) float switch sensor, and (**i**) flow sensor. A breadboard was used for circuiting (instead of a printed circuit board) to be able to easily change or replace components of the system in the field.

**Figure 2:**
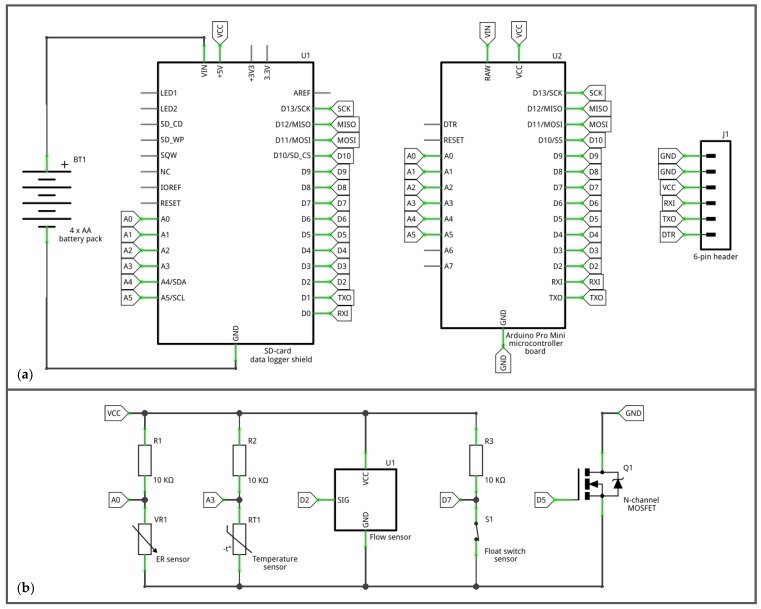
Circuit diagram of the multi-sensor monitoring system: (**a**) the microcontroller board and data logger shield combination and (**b**) the sensors. For the circuit reference designator list, see Appendix A.

The multi-sensor monitoring system consists of a microcontroller board combined with a data logger shield, and four sensors: an electrical resistance (ER) sensor, temperature sensor, float switch sensor, and flow sensor (Figure 1 and Figure 2). The microcontroller board and data logger shield combination function as a data logger. The ER sensor, temperature sensor, and float switch sensor provide information on the presence of water, while the flow sensor provides information on the occurrence of flow. The sensors were selected based on initial tests in the lab and the system was tested during two field seasons (2016 and 2017). The combined price of the parts of the monitoring system, including the field installation materials, is around 80 US dollars.

### 2.1. Microcontroller Board and Data Logger Shield Combination 

The microcontroller board used to operate the multi-sensor monitoring system is the open-source Arduino Pro Mini (5 V model) (Arduino, New York City, NY, USA) (Figure 1). The Pro Mini is based on the ATMega328 microcontroller (Atmel, San Jose, CA, USA), which runs at 16 MHz (facilitated by an on-board oscillator) and provides the board with 32 KB flash memory, 2 KB SRAM, and 1 KB EEPROM. The board has 14 digital in/output pins and six analog pins to which sensors and actuators can be connected. All pins have access to an internal pull-up resistor of 20–50 KΩ. Two of the digital pins can be used as external interrupt pins, which allow an external signal on the pins to interrupt the processor and start a separate piece of code. The six analog pins all have 10-bit analog to digital converters (ADC), which convert analog voltage signals into discrete analog levels between 0–1023 (ADC values). Although the Pro Mini operates at 5 V DC, it can accept voltage up to 12 V DC, because of the voltage regulator on the board. The board draws about 15 mA and was chosen over other boards, such as the standard Arduino Uno, because of its relatively low power consumption (see Section 2.3 for more details on the power consumption of the monitoring system).

For data logging, the Arduino Pro Mini was combined with an SD-card data logger shield (Adafruit Industries, New York City, NY, USA) (Figure 1). The data logger shield integrates an SD-card interface with a real-time clock (RTC), and includes a prototyping area. The SD-card interface allows data to be saved on FAT16 or FAT32 formatted SD-cards and the RTC can be used to provide the saved data with a time stamp. The RTC is powered by a 3 V lithium coin cell battery, which ensures that it keeps running even when the shield is not powered on. The prototyping area consists of a grid with 2.5 mm diameter holes and permits extra circuiting. The data logger shield was originally designed to be used with the Arduino Uno (or similar board), but since the Pro Mini uses the same microcontroller and has the same pins necessary to operate the shield, it is electronically equally compatible. However, because the Pro Mini is smaller than the Uno and has a different pin layout, the shield cannot be stacked on top of the board (which is possible for the UNO). To connect the Pro Mini to the shield, the board was therefore soldered onto the prototyping area of the shield and from there the pins of the Pro Mini were wired to the corresponding pins of the shield.

The microcontroller board and data logger combination was programmed as an interval logger that logs the sensor data every 5 min (see Section 2.4 for more details on the custom-written operating program for the multi-sensor monitoring system). The data was written on a 2 GB SD-card. Because each data log only used 60 bit, this setup allowed for years of data storage.

The microcontroller board and data logger combination was programmed using the Arduino Integrated Development Environment (IDE) software. The software enables writing sketches (programs) and uploading them to the microcontroller board. Sketches are written in a curtailed version of the programming language C++, and stored in the flash memory of the microcontroller. To upload sketches, the board was connected to a computer using a six-pin header (which was soldered onto the programming header of the board, see Figure 1), a FT23RL chip (Future Technology Devices International, Glasgow, UK) based breakout board (SparkFun Electronics, Niwot, CO, USA), and a USB cable with USB A and USB mini B male connections.

### 2.2. Sensors

**Figure 3:**
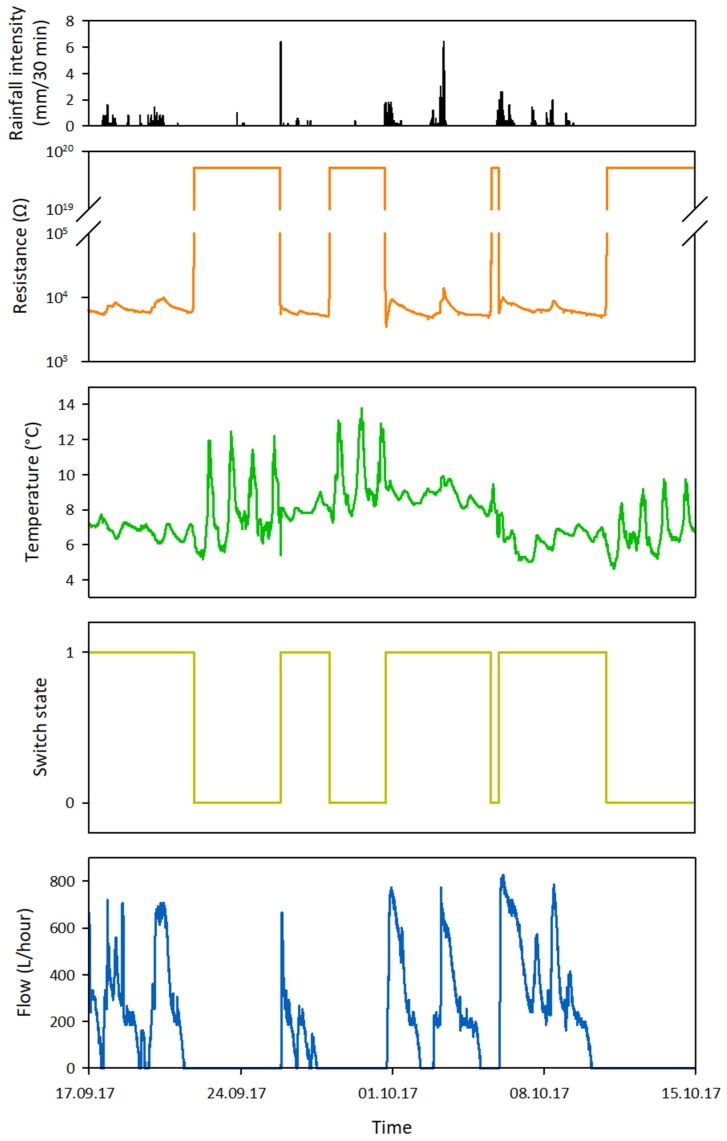
An example of four weeks of raw sensor data for the multi-sensor monitoring system together with the 30-minute rainfall intensity during this period.

**Figure 4:**
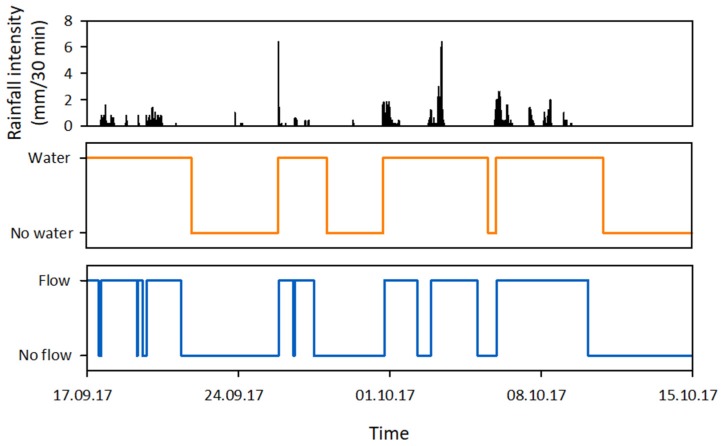
An example of four weeks of processed sensor data for the ER sensor and flow sensor together with the 30-minute rainfall intensity during this period.

Nine low-cost sensors that had the potential to provide either information on the presence of water or the occurrence of flow were evaluated during initial lab tests (see Supplementary Material, Description S1, and Tables S1 and S2 for a general description and the results of these initial lab tests). Of these sensors, the ER sensor, temperature sensor, float switch sensor, and flow sensor were considered suitable for further testing in the field. During the field tests, raw sensor data (Figure 3) was collected (see Section 3 for more details on the field tests). This data was converted into hydrological state data (Figure 4), which was then used to evaluate the sensor performance (see Section 4.2 for more details on the evaluation of the sensor performance). Prior to the second field season, modifications were made to the original design of some of the sensors to improve their robustness and sensitivity.

#### 2.2.1. ER Sensor

The ER sensor (Figure 1) consists of two single-core copper wires (1.8 mm diameter) with polyvinyl chloride (PVC) insulation that is stripped off (50 mm) at the end of the wires to form two sturdy electrodes. The ER sensor provides information about the presence and absence of water by measuring the electrical resistance between the electrodes [cf. 35,46,48]. The resistance is generally low when water is present and high when water is absent. 

Similar to Blasch et al. (2002) [46] and Bhamjee and Lindsay (2011) [35], the ER sensor design does not include a housing for the electrodes. However, to minimize the number of false positives (incorrect water states in the state data derived from the sensor) related to damp sediment on the electrodes, the electrodes were made significantly longer than in these previous studies. Longer electrodes reduce the chance of the electrodes being completely covered by damp sediment, and it is relatively easy to distinguish a damp sediment signal from a water signal in the resistance data when electrodes are only partly covered by sediment. As additional measures, the electrodes were shielded and installed slightly above the streambed, similar to the setup of Bhamjee and Lindsay (2011) [35] (see Section 3.2 for more details on the field setup of the ER sensor).

To measure the resistance between the electrodes, the ER sensor was connected to the microcontroller board and data logger shield combination using the setup shown in Figure 2. This setup creates a voltage divider that allows the voltage drop over the 10 KΩ resistor to be measured using the analog pin. The voltage drop over the 10 KΩ resistor changes when the resistance between the electrodes of the ER sensor changes. The 10-bit ADC on the analog pin converts the measured voltage signal into an ADC value between 0–1023. The microcontroller then calculates the resistance (Ω) between the electrodes using a rearrangement of the voltage divider equation that incorporates the ADC value:

R = R_r_ / (1023 / ADCvalue − 1), (1)

where R is the resistance (Ω) between the electrodes and R_r_ the resistance of the resistor (10 KΩ). 

The raw resistance data (Figure 3) was converted into state data (Figure 4) by assigning a catchment specific filter to the data. The filter was based on information for the upper and lower boundaries of the resistance ranges for wet and dry channel conditions for the field test site, and on the observed changes in resistance for channel wetting and drying sequences. 

To determine the upper and lower boundaries of the resistance range for wet channel conditions, the resistance was measured for two solutions, with electrical conductivities of 30 µS/cm and 380 µS/cm, respectively. These solutions represent the typical minimum and maximum EC of stream water for the field test site [cf. 48]. To determine the upper and lower boundaries of the resistance range for dry channel conditions, the resistance was measured for ‘free’ electrodes (no water, no sediment) and for electrodes covered with damp sediment (where the sediment was wetted using the solution that represented the maximum stream water EC). The measurements were repeated for four different ER sensors to account for variability between the sensors. The measurements for free electrodes gave ADC values of 1023 and therefore infinite resistance according to Equation (1). Since the resistance of air is not infinite, the actual resistance for free electrodes was determined based on the electrical resistivity equation:

R = ρ · L / A,(2)

where R is the resistance between the electrodes (Ω), ρ the average resistivity of air (3.2 × 10^19^ Ω·mm), L the distance between the electrodes (70 mm) and A the surface area of the electrodes (30.8 mm^2^).

The resistance for wet channel conditions ranged from 1.1 × 10^3^ to 1.7 × 10^4^ Ω and for dry channel conditions from 8.7 × 10^3^ to 5.2 × 10^19^ Ω. The ranges thus partly overlap. Applying a simple threshold filter to convert the resistance data into state data, as was done in previous studies [35,48], would therefore lead to incorrect state data. 

**Figure 5:**
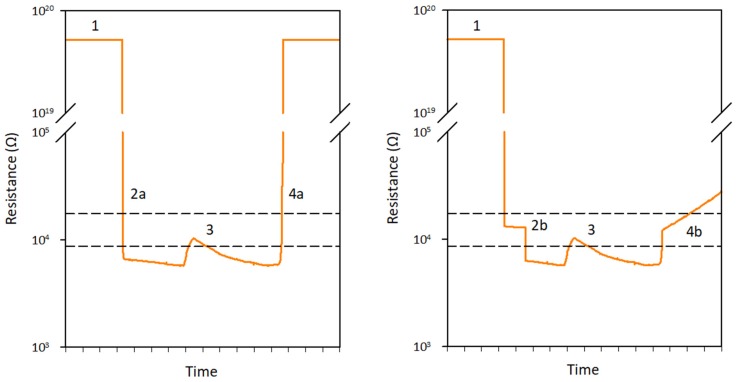
Examples of the typical changes in resistance for channel wetting and drying sequences. The resistance signals indicate: (1) a dry channel, (2a) general wetting of the channel, (2b) wetting of the channel including the formation of rainfall puddles around the sensor, (3) a wet channel, including dilution of the stream water during a rainfall event, (4a) general drying of the channel, and (4b) drying of the channel with damp sediment on the electrodes. Sequences that combine signals 2a and 4b, and 2b and 4a were also observed. The dashed lines indicate the upper and lower boundaries of the overlap of the wet and dry channel resistance ranges. For resistance signals within the overlap range, resistance signals 2b and 3 were assigned a water state and 4b a no water state. Values above the upper boundary (>1.7 × 10^4^ Ω) were assigned a no water state and values below the lower boundary (<8.7 × 10^3^ Ω) a water state.

The strategy for converting values within the overlap range was based on the typical changes in resistance that were observed for channel wetting and drying sequences (Figure 5):

1. When the channel was dry, the resistance was higher than the overlap range (#1 in Figure 5).
2. When the channel wetted up, the resistance generally dropped instantly to below the overlap range (#2a in Figure 5). In some cases, the resistance instead dropped to within the overlap range and then levelled out for some time, before instantly dropping for a second time to below the overlap range (#2b in Figure 5). This indicates wetting of the channel including rainfall puddles forming around the sensor.
3. When the channel was wet, the resistance signal was generally stable and remained below the overlap range. However, sometimes the signal rose and peaked within the overlap range (#3 in Figure 5). This indicates dilution of the stream water (lowering of the EC) during rainfall events.
4. When the channel dried up, the resistance generally rose instantly to above the overlap range (#4a in Figure 5). However, in some cases the signal instead showed a quick rise to within the overlap range, followed by a more gradual increase (#4b in Figure 5). This indicates the gradual drying of damp sediment on the electrodes.

Three resistance signals of the channel wetting and drying sequence fall within the overlap range: the signal caused by rainfall puddles, the signal that indicates the dilution of stream water during rainfall events, and the signal of damp sediment on the electrodes. The first two correspond to water states and the last one to a no water state. Since the shape of the last signal is easily distinguishable from the first two, the data in the overlap range could be converted into water and no water states based on the shape of the signal. Values outside of the overlap range were converted into water and no water states using the upper and lower boundaries of the overlap range (R < 8.7 × 10^3^ Ω = water and R > 1.7 × 10^4^ Ω = no water).

#### 2.2.2. Temperature Sensor 

The temperature sensor (Adafruit Industries, New York City, NY, USA) (Figure 1) consists of a thermistor (thermal resistor, length 10 mm), coated with epoxy to make it waterproof and robust. The temperature sensor can provide information about the presence and absence of water, because the amplitude of the diurnal temperature signal for water is smaller than for air [cf. 44,45]. The temperature is determined by measuring the resistance of the thermistor and converting it into temperature. The thermistor is a negative temperature coefficient (NTC) type thermistor, meaning that the resistance of the thermistor decreases as the temperature increases. The resistance of the thermistor at 25 °C is 10 KΩ (+/− 1%). The accuracy and the precision of the sensor are 0.25 °C and 0.01 °C, respectively. 

To measure the temperature, the sensor was connected to the microcontroller board and data logger shield combination using the setup shown in Figure 2. This setup is the same as the ER sensor setup and allows the resistance of the thermistor to be measured using the same principles and equation as were used to measure the resistance between the electrodes of the ER sensor. The microcontroller then calculates the temperature (°K) by converting the resistance of the thermistor into temperature using the B parameter equation based on the Steinhart–Hart equation [52]:

1 / T = (1 / T_0_) + (1 / B) · ln(R / R_0_), (3)

where T is the temperature (°K), T_0_ the room temperature (25 °C = 298.15 °K), B the thermistor coefficient (3950 °K), R the resistance of the thermistor (Ω), and R_0_ the resistance of the thermistor at room temperature (10 KΩ). In a final step, the microcontroller converts the temperature from degrees Kelvin into degrees Celsius. 

The raw temperature data (Figure 3) was converted into state data by assigning a catchment specific filter to the data. The filter was largely based on the moving standard deviation technique introduced by Blasch et al. (2004) [45]. This technique determines water/no water states by applying a moving standard deviation filter to the temperature data. The advantage of using the moving standard deviation of temperature is that it amplifies short-term variations and removes long-term fluctuations. The filter is based on five parameters: the length of the moving standard deviation window, the reference timing within the window, the state change threshold, the minimum duration of a water state, and the minimum duration of a no water state. 

The parameters were determined by comparing the moving standard deviation temperature data of four representative monitoring locations for the field test site with the state data of the ER sensors from these locations (data from the 2016 field season). The window length was chosen from a range between 30 min and 6 h, while the reference timing was set at either the beginning, center, or end of the window. Based on a visual examination of all combinations, a one-hour window with a centered reference timing was considered optimal, because this provided the clearest distinction between water/no water states and the most accurate state change timing.

**Figure 6:**
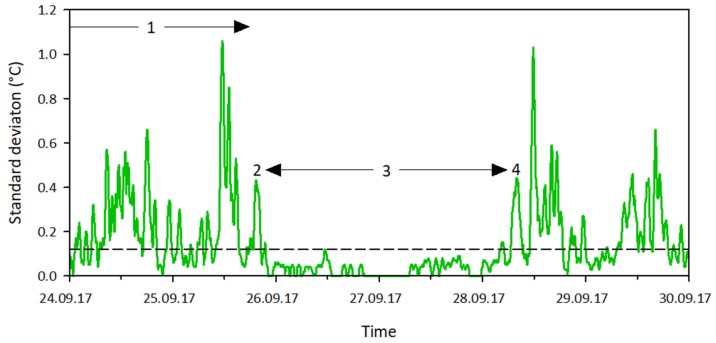
Example of the typical changes in the moving standard deviation of the temperature for wetting and drying sequences. Signals (**1**) and (**3**) indicate a dry and a wet channel respectively. A state change from a dry to wet channel (**2**) is indicated by a peak in the moving standard deviation with a maximum of at least 0.20 °C, followed by a dip below the 0.12 °C threshold (dashed line) for at least 2.5 h. A state change from wet to dry channel (**4**) is indicated by a rise in the moving standard deviation above the 0.12 °C threshold, followed by a peak with a maximum of at least 0.20 °C, and a stay above the threshold for at least 3 h. The timing of both state changes coincides with the peaks.

Using this window length and reference timing, the state change threshold was determined. This was done by examining the typical changes in the moving standard deviation of the temperature for channel wetting and drying sequences (Figure 6):

1. When the channel was dry, the moving standard deviation was larger than 0.12 °C during daytime and often smaller than 0.12 °C during nighttime (#1 in Figure 6).
2. When the channel wetted up, the moving standard deviation first peaked with a maximum of at least 0.20 °C and then the signal dropped below 0.12 °C. The timing of the peak coincided with the timing of the state change (#2 in Figure 6).
3. When the channel was wet, the moving standard deviation remained relatively stable and below 0.12 °C (#3 in Figure 6).
4. When the channel dried up, the moving standard deviation first increased to above 0.12 °C and then peaked with a maximum of at least 0.20 °C. The timing of the peak coincided with the timing of the state change (#4 in Figure 6).

The sequence shows that the prerequisite for a state change involves the standard deviation signal crossing a 0.12 °C threshold preceded (in case of channel wetting) or followed (in case of channel drying) by a peak in the signal with a maximum of at least 0.20 °C. It further shows that the state change timing coincides with this peak. Merely applying a threshold value to the data for the state change timing, as was done in the study by Blasch et al. (2004) [45], would therefore lead to incorrectly timed state changes and to false water states during nighttime. 

Finally, the minimum duration of water and no water states were determined by comparing the true/false state ratios for minimum state durations ranging from 30 min to 4 h. A 2.5 h minimum duration for water states and a 3 h minimum duration for no water states achieved the best true/false state ratio. Examination of the data showed that false states were more likely to occur at the transition from daytime to nighttime or vice versa. To further improve the true/false state ratio, two additional conditions were therefore included:

1. A water state that started during daytime and ended in the subsequent night or vice versa, needed to include a minimum of 2.5 h of daytime.
2. A no water state that started during daytime and ended in the subsequent night or vice versa, needed to include a minimum of 3 h of daytime.

#### 2.2.3. Float Switch Sensor

The float switch sensor (Hamlin Electronics L.P., Lake Mills, WI, USA) (Figure 1) consists of a cylindrical polypropylene (PP) blown float (height 16 mm, diameter 23 mm) which slides along a PP vertical stem (height 44 mm, diameter 5.5 mm). The float has a ring magnet encased in its lower end. The stem contains a hermetically sealed magnetic reed switch circuit and has an integral M8 × 1.25 mm pitch thread connection (length 12 mm) at the top. A hexagonal platform (diameter 12 mm) below the pitch thread and a clip-on platform (diameter 20 mm) at the bottom of the stem prevent the float from sliding off the stem. 

The float switch sensor provides information about the presence and absence of water by measuring the state of the reed switch [cf. 49]. When the water level rises or falls, the float moves up or down the vertical stem, causing the magnet in the float to open or close the reed switch. The float switch sensor used in this study is a SPST-NC (single pole, single throw, normally closed) type switch, meaning that when the float moves up the vertical stem and the magnet is aligned with the reed switch, it causes the contacts of the switch circuit to open. The water level offset required for the float to open the reed switch is 1 cm. 

To protect the float switch sensor from sediment and debris, a housing was added to the sensor. During the first field season, the float switch sensor was housed in a PVC pipe (height 300 mm, diameter 50 mm) with six slits (length 50 mm, width 2 mm) at its lower end, to allow the inflow of water. However, with this setup, the sensor often failed to switch on time or sometimes did not switch at all during the wetting and drying of the channel (see Section 4.2 for more details on the performance of the float switch sensor). This was primarily caused by the sensor getting stuck in the pipe, and by fine sediment and organic matter settling on the clip-on platform, which prevented the float from moving all the way down the vertical stem. 

**Figure 7:**
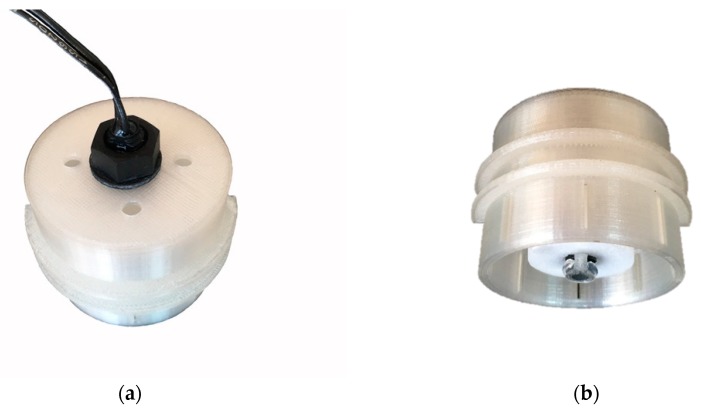
3D-printed PLA (polylactide) housing for the float sensor, with the sensor inside and the float resting on the 3D-printed PLA platform: (**a**) top/side view and (**b**) bottom/side view.

To solve these problems, the design of the float switch sensor was modified after the first field season. To prevent the sensor from getting stuck in the housing, the PVC pipe was replaced with a custom-designed, 3D-printed, polylactide (PLA) housing (height 35 mm, diameter 44 mm) (Figure 7) (Supplementary Material, 3D-print object S1) that could be screwed onto the integral thread of the vertical stem to securely fix the position of the sensor in the housing. The housing generally resembles the PVC cap used in the study by Mcdonough et al. (2015) [49], but in addition to being open at the bottom, it has an additional eight slits (length 10 mm, width 0.5 mm) at its lower end to ensure a sufficient inflow of water in case of obstruction at the bottom. Furthermore, four air holes were added to the roof of the housing to prevent air bubbles from being trapped inside the housing and restricting the float from moving upward when the water level rises. To minimize the chance of fine sediment accumulating on the platform and restricting the float from moving down, the clip-on ring platform was replaced with a custom-designed, 3D-printed, PLA platform (Figure 7) (Supplementary Material, 3D-print object S2) with a surface area 10 times smaller than that of the clip-on ring platform. Additionally, the housing was covered with a filter sock.

To measure the state of the reed switch, the float switch sensor was connected to the microcontroller board and data logger shield combination using the setup shown in Figure 2. In this setup, the 10 KΩ resistor serves as a pull-up resistor that ensures that the digital pin measures a high state (1) when the reed switch is open and a low state (0) when the reed switch is closed. Additionally, the resistor prevents a short circuit when the reed switch is closed. The use of an external 10 KΩ pull-up resistor was preferred over the relatively high impedance (20–50 KΩ) internal pull up resistors of the microcontroller board, because a high impedance pull-up resistor in combination with long wires (as used in the field setup) makes the digital pin more susceptible to electromagnetic interference.

#### 2.2.4. Flow Sensor

The flow sensor (YIFA Plastic Products Co., ltd, Yuè, Foshan, China) (Figure 1) consists of a 66% nylon + 33% glass fiber valve body, a polyoxymethylene (POM) impeller, and a Hall-effect sensor. The valve body consists of a main chamber with an integral male pipe thread connection on both sides through which water enters and exits the sensor. The impeller is equipped with an integrated ring magnet with alternating zones of polarity and is situated in the main chamber. The Hall-effect sensor is situated in an adjacent waterproof compartment. 

The flow sensor provides information on the occurrence of flow by measuring the pulse output of the Hall-effect sensor. When water flows through the valve body, the impeller spins and moves the ring magnet past the Hall-effect sensor. The alternating magnetic fields of the ring magnet cause the Hall-effect sensor to switch between a high state (ON) (closed circuit) and low state (OFF) (open circuit). The rate of the resulting pulse signal can be converted into discharge. The Hall-effect sensor used in this flow sensor is a latching switch type. This type typically switches to an ON state when subjected to a positive magnetic field and to an OFF state when exposed to a negative magnetic field, and latches the state until an opposite magnetic field is presented. 

**Figure 8:**
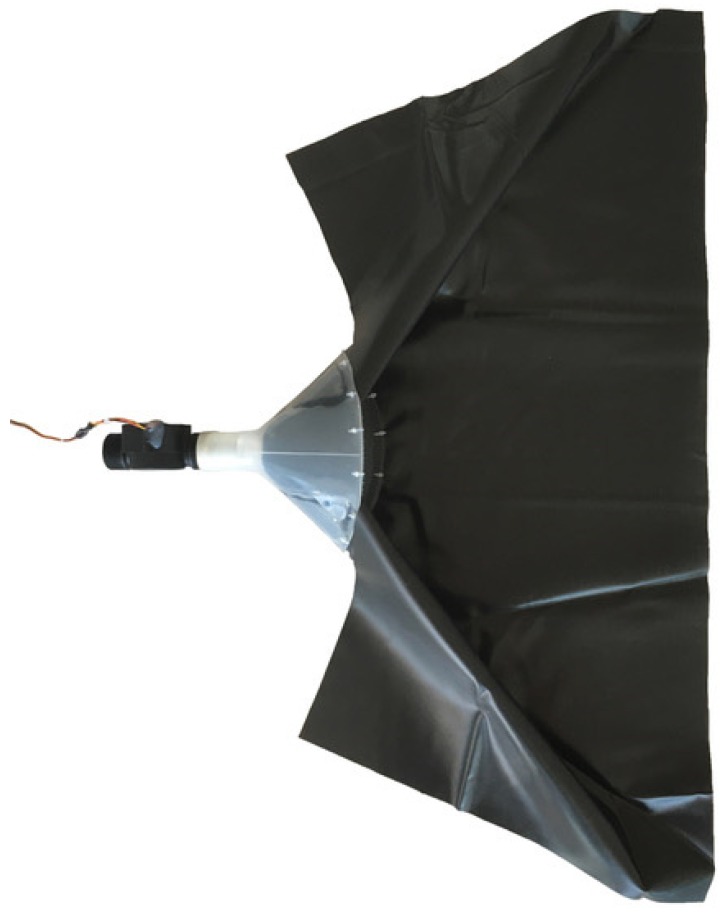
Top view of the flow sensor with funnel and tarp (2017 field season setup).

To direct water into the flow sensor, a PP funnel with a piece of tarp attached to the funnel mouth was connected to the flow sensor (Figure 8). The funnel (length 200 mm, mouth diameter 220 mm, neck diameter 45 mm) was flattened on one side (after placing it in a hot air oven) to allow it to be positioned flat on the channel bed. The funnel mouth was covered with polyethylene (PE) mesh (mesh size 3×3 mm) to prevent coarse sediment from entering the valve body and blocking the impeller. The tarp (length 40 cm, width 100 cm) is a flexible extension of the funnel, which can cover (most of) the wetted perimeter of small channels (see Section 3.2 for more details on the field setup of the flow sensor). In the case of high flows, water not only flows through but also over the funnel. However, since the sensor is used to provide information on flow/no-flow states and not on the actual discharge, this is not an issue.

During the first field season, the G1 version of the flow sensor (length 114 mm, width 49 mm, height 71 mm, major diameter of pipe thread connection 33 mm, flow rate range 1–100 L/min) was used. The flow sensor was connected to the neck of the funnel using a PVC pipe cap (diameter 45 mm) with a hole (diameter 32.5 mm) drilled in its top. The cap was plugged into the neck of the funnel and the flow sensor was screwed into the hole of the cap using the pipe thread connection. The tarp used in the setup was a polytarp, and was attached to the funnel mouth using nylon tie-wrap cables. However, with this setup, the flow sensor was not able to consistently detect low flows (that where within the flow range of the sensor), which resulted in a little less than half of the recorded state changes to be timed incorrectly (see Section 4.2 for more details on the performance of the flow sensor). This was primarily attributed to the use of the PVC cap to connect the flow sensor to the funnel, which did not allow optimal alignment of the flow sensor and the funnel and therefore resulted in the impeller not being consistently activated during low flows. Other issues included degradation of the tarp and too much water ponding in front of the funnel. 

To solve these problems, the setup was slightly modified after the first field season. To improve the alignment of the flow sensor and the funnel, the PVC cap was replaced by a custom-designed, 3D-printed, PLA pipe fitting (Supplementary Material, 3D-print object S3) with a female pipe thread connection that is compatible with the male pipe thread of the flow sensor. To reduce ponding in front of the funnel, the G1 flow sensor was replaced by the larger G5/4 version (length 130 mm, width 51 mm, height 74 mm, major diameter of pipe thread connection 42 mm, flow rate range of 1–120 L/min). Since ponding is a natural phenomenon in the step-pool streams of the field test site, some ponding was, however, considered acceptable. The polytarp was replaced by a sturdier, UV resistant, soft PVC pond liner, which was glued onto the funnel for a more robust setup.

To measure the discharge, the flow sensor was connected to an interrupt pin (digital pin 2) on the microcontroller board (Figure 2). When water flows through the flow sensor and the impeller spins the ring magnet past the Hall-effect sensor, the state of the interrupt pin switches between high and low states. The microcontroller then counts the number of changes from low to high states (pulses) for a three-second interval and subsequently converts this number into discharge (L/hour) using the following equation:

Q = (N / 3) · 60 · F_c_, (4)

where Q is the discharge (L/hour), N the number of pulses and F_c_ the flow coefficient (pulses/second for one L/min discharge) of the flow sensor. The F_c_ is 5.5 pulses/second for one L/min discharge for the G1 version of the flow sensor, and 4.5 pulses/second for one L/min discharge for the G5/4 version.

**Figure 9:**
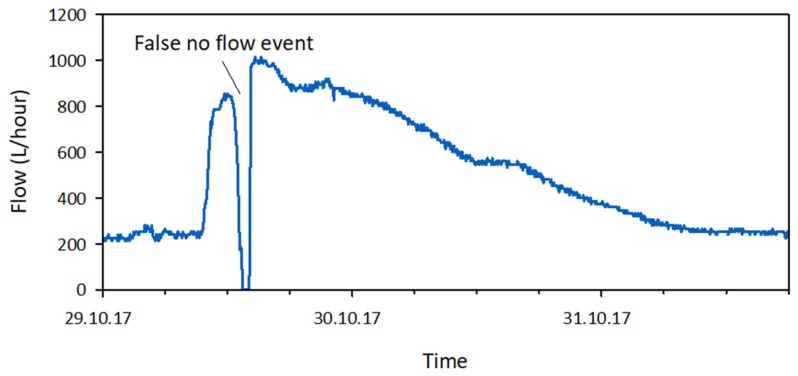
Example of a false no-flow event in the discharge data, due to temporary clogging of the funnel of the flow sensor during stormflow.

The raw discharge data (Figure 3) was converted into state data (Figure 4) using a simple threshold filter. Discharge values higher than zero were assigned a flow state and discharge values of zero were assigned a no-flow state. At some monitoring locations on the field test site, the funnel of the flow sensor temporarily got clogged during peak flow conditions for some of the largest events. This resulted in ‘gaps’ in the data with discharge values of zero (Figure 9). These false no-flow events were filtered from the discharge data based on the shape of the signal and assigned a flow state.

### 2.3. Power Saving Measures

During the initial lab tests, the setup consisted of a standard Arduino Uno microcontroller board, the SD-card data logger shield, and the sensors that were being tested. The setup was powered using an AC-to-DC adapter. For the setup with the ER sensor, temperature sensor, float switch sensor, and flow sensor, the measured current draw was 55.3 mA during data logs, and 52.3 mA in between data logs. This power consumption was too high to allow long-term collection of high temporal resolution data when using regular size batteries to power the system. Therefore, several measures (based on suggestions on the Arduino Forum [53]) were taken to lower the current draw drastically (Table 1):

1. Using the Arduino Pro Mini

As mentioned before, the Arduino Pro Mini microcontroller board was used instead of the standard Arduino Uno in the final design of the multi-sensor monitoring system. Although the Pro Mini is based on the same microcontroller and has the same number of digital and analog pins as the Uno, the Pro Mini does not have a USB host, barrel jack connection and several other peripherals. Therefore, it consumes considerably less power. Using the Pro Mini instead of the Uno saved 33.0 mA in current draw. 

2. Powering down the microcontroller and other on-board peripherals in between data logs

When the Arduino Pro Mini is running normally, the on-board peripherals including the ATmega328 microcontroller, ADC, Brownout Detection (BOD), external reset, Inter-Integrated Circuit (I^2^C), Serial Peripheral Interface (SPI), Universal Synchronous/Asynchronous Receiver-Transmitter (USART), and Watchdog Timer (WDT) all consume power. To save power, the Pro Mini was programmed to power down the microcontroller and the other on-board peripherals (except for the external reset and WDT) in between data logs, using the functions sleep.pwrDownMode and sleep.sleepDelay from the Library Sleep_n0m1 (NoMi Design Ltd.). This saved 14.8 mA in current draw in between data logs.

3. Powering down the sensors in between data logs

As the sensors only require power when they are being read, they can be powered down in between data logs. This was done using a logic level, N-channel, metal-oxide-semiconductor field-effect transistor (MOSFET) (type IRLB8721PbF, Infineon Technologies Americas Corp. El Segundo, CA, USA) controlled by a digital pin on the Arduino Pro Mini (Figure 1 and Figure 2). The use of a transistor was preferred over using a digital pin directly, because even though the general current draw of the sensors was within the range of the digital pins on the Pro Mini, the initial current draw to charge the capacitance of the sensors could exceed the maximum current rating of the pin and as a result damage it. A transistor, on the other hand, can supply power to the sensors from the Vcc pin and therefore supply more current for this initial charge. Although slightly more expensive, a MOSFET was preferred over a bipolar junction transistor (BJT), because MOSFETs are generally more power-efficient. Ultimately, this measure saved 3.4 mA in current draw in between data logs.

4. Removing the power LEDs

Both the Arduino Pro Mini and the SD-card data logger shield have power LEDs that are turned on when the board and shield are running, even in power-down mode. Because these LEDs only serve to indicate that the board and shield are powered on, they were considered redundant and therefore de-soldered from the board and shield. This saved 1.9 mA in current draw.

In total, the power saving measures reduced the current draw to 20.4 mA during data logs and 0.2 mA in between data logs. Since data logs only take three seconds, the multi-sensor monitoring system ran on 0.2 mA for most of the time.

During the field tests, the system was powered by four Energizer L91 lithium AA batteries (Energizer Holdings, Inc. St. Louis, MO, USA). These batteries were chosen because, next to their practical seize and weight, they have a relatively high capacity (3200 mAh) and perform well in outdoor conditions.

### 2.4. Operating Program

The program to run the multi-sensor monitoring system (Supplementary Material, Arduino sketch S1) largely follows the general structure of the Arduino programming language, and was partly based on code provided by the Adafruit Learning System [54].

In the first part of the program, the libraries are included, the pins are defined and the global variables and objects are declared. For the monitoring system, these are:

- The libraries used for the communication of the microcontroller with the SD-card and RTC, and the one used to power down the microcontroller and other on-board peripherals
- The pins used to select the SD-card, control the MOSFET and read the sensors
- The variables related to the sensor output conversion and the power-down interval
- The log file, RTC and power-down objects

The second part consists of two functions:

- The error function, which is called when something is wrong with the SD-card, and then prints the type of error to the Serial Monitor.
- The interrupt function named flow, which is called when the interrupt pin connected to the flow sensor measures a change from a low to a high state, and then counts the number of pulses.

The third part is the setup function, which is called when the microcontroller board is powered on and executes a series of tasks that only have to be executed once, at the start of the program. For the monitoring system, these tasks include:

- Initializing digital pin modes (INPUT for the sensors and OUTPUT for the SD-card select and the MOSFET)
- Setting up the interrupt pin for the flow sensor
- Initializing the SD-card and RTC
- Creating a log file with headers

The last part is the loop function, which executes a series of tasks over and over until the microcontroller board is turned off. For the monitoring system, these tasks include in consecutive order:

- Powering on the microcontroller and other on-board peripherals
- Powering on the sensors
- Obtaining the current date and time from the RTC
- Logging the date and time
- Reading, converting and logging the sensor output
- Writing the data to the SD-card
- Powering down the sensors
- Powering down the microcontroller and other on-board peripherals
- Remaining in power-down mode for the duration of the power down interval

## 3. Field Test 

### 3.1. Study Site 

**Figure 10:**
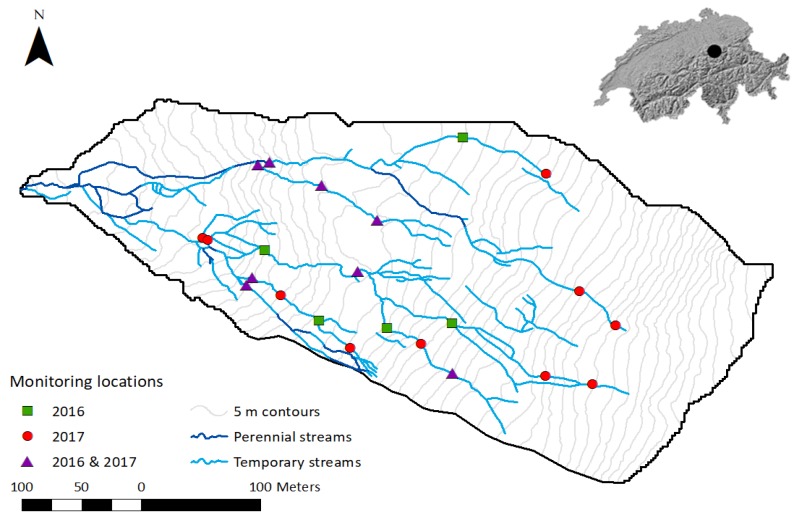
Map of the field test site, including all field-mapped streams and the monitoring locations, where the multi-sensor monitoring system and time-lapse cameras were installed. Some locations were used exclusively during the 2016 field season (green squares) or the 2017 field season (red circles), the others were used during both seasons (purple triangles). The inset map indicates the location of the field test site (black dot) within Switzerland.

The multi-sensor monitoring system was tested in a small mountainous headwater catchment in Switzerland (Figure 10) in the summer and fall of 2016 and 2017. The 0.12 km^2^ catchment is situated in the Alptal watershed. The catchment elevation ranges from 1421 to 1656 m.a.s.l. and the topography is characterized by alternating steep slopes (>20°) and flatter areas, caused by landslides and soil creep. The catchment is covered by forest (mostly spruce), open forest, meadows, and wetlands [55]. The bedrock consists of relatively impermeable Tertiary Flysch, consisting of layers of calcareous sandstone, marl and schist, and argillite and bentonite schists [56,57]. The soils on the steep slopes, where the groundwater level is generally more than 40 cm below the soil surface, are umbric Gleysols. In the flatter areas, where the groundwater level is generally close to the soil surface, the soils are mollic Gleysols. Soil depth ranges from 0.5 m on the steep slopes to 2.5 m in the flatter areas. The climate is humid, with a mean annual temperature of 6 °C [56] and a mean annual precipitation of 2300 mm [58]. Despite the relatively high precipitation input, most streams in the catchment are temporary. The temporary stream regimes range from quasi-perennial to episodic (based on the regime classification by Gallart et al. (2017) [2]). In the episodic reaches, flow during rainfall events typically lasts several hours. The streams are generally small (bankfull width: 10–200 cm, bankfull depth 15–60 cm) with a step-pool character, but differ significantly in width/depth ratios, entrenchment ratios (flood-prone width divided by bankfull width), bed material and slope. Stream mapping in the summer and fall of 2015 showed that the drainage density can increase by a factor of five between dry periods and rainfall events [59,60]. The discharge at the outlet ranged between 1 and 140 L/s during the two field seasons. The large variety in temporary stream regimes and characteristics allowed the monitoring system to be tested in a large range of settings.

### 3.2. Multi-Sensor Monitoring System Setup

**Figure 11:**
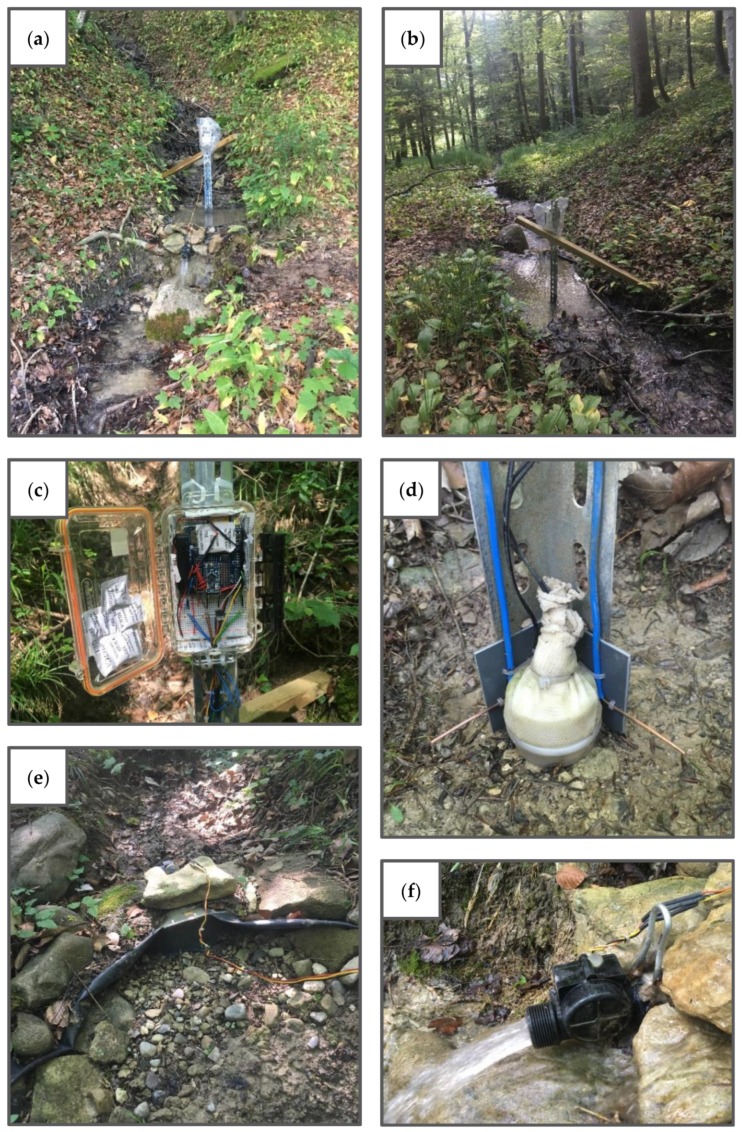
Field setup of the multi-sensor monitoring system (2017 field season): (**a**) upstream view of the monitoring system in a flowing stream, (**b**) downstream view of the monitoring system in a flowing stream, (**c**) the waterproof box, containing the microcontroller board and data logger shield combination, battery pack and MOSFET, attached to the top of the angle bar, (**d**) the ER sensor, float switch sensor (wrapped in a filter sock) and temperature sensor (in a sheltered pocket behind the float switch sensor) attached to the angle bar (using an angled PVC sheet) at streambed level, in a dry stream (**e**) downstream view of the flow sensor setup in a dry stream, including the tarp buried into the channel bed and bank (**f**) the flow sensor during a flow event, and the double legged peg that secures the funnel neck to the channel bed.

The multi-sensor monitoring system was installed at 13 locations in the stream network during the 2016 field season and at 18 locations during the 2017 field season (Figure 10). The setup was similar at every location (see example in Figure 11) and consisted of:

- a slotted steel angle bar (length 105 cm, side width 3.5 cm and angle 90°)
- a wooden crossbar (length 150 cm)
- a waterproof box (length 18.5 cm, width 11 cm, height 4.5 cm) containing the microcontroller board and data logger shield combination, battery pack and MOSFET
- the sensors

The angle bar was hammered into the streambed with its angle pointing upstream and secured to the crossbar, which was hammered into the stream bank. The waterproof box was attached to the top of the angle bar, on the leeside of the angle. The sensors were connected to the circuitry inside the box through a hole (with a rubber grommet) in the lower end of the box. The ER sensor, temperature sensor, and float switch sensor were attached to the angle bar at streambed level, on the leeside of the angle. The flow sensor was installed 30 to 70 cm (depending on the channel size) downstream from the angle bar and the other sensors.

The ER sensor, temperature sensor, and float switch sensor were attached to the angle bar using an angled PVC sheet (length 5.5 cm, side width 4.5 cm and angle 90°). The sheet allowed the sensors to be securely fixed to the bar and acted as a buffer between the sensors and the bar to avoid electric and heat conduction. Because the switch offset for the float switch sensor is 1 cm, the ER sensor and temperature sensor were installed 1 cm above the streambed. This simultaneously reduced the chance of sediment accumulation on the sensors. The electrodes of the ER sensor were positioned in line with the sides of the angle bar to further reduce sediment buildup around the sensor. During the 2016 field season, the temperature sensor was positioned on the downstream side of the float switch sensor. During the 2017 field season, the temperature sensor was positioned in a sheltered pocket in between the float switch sensor and the angle bar to reduce the chance of sediment buildup around the sensor, and thus, improve the ability of the sensor to provide correctly timed state changes (see Section 4.2 for more details on the performance of the temperature sensor).

The flow sensor was installed in the channel by securing the funnel to the channel bed and burying the tarp into the channel bed and banks. The funnel was secured to the bed at the funnel neck using a double-legged peg (25 cm). Additionally, several heavy stones were placed on top of the funnel. To install the tarp, first, a layer of 5–10 cm of sediment was removed from the bed and banks, then the tarp was spread out and the edges were fixed to the bed and banks using 12 cm stainless steel nails, and finally, the tarp was covered with the initially removed bed and bank material. 

### 3.3. Time-Lapse Cameras

To evaluate the performance of the sensors, time-lapse cameras were installed at all monitoring locations (Figure 10). The camera used was the Bushnell Trophy Cam (model 119437C, Bushnell Outdoor Products, Overland Park, KS, USA), which is a trail camera with a time-lapse function. The camera is rain and snow resistant and has built-in infrared LEDs that are used as a flash and allow the camera to take clear photos during nighttime. The camera runs on eight AA batteries.

The setup was similar at every monitoring location. A similar angle bar as was used in the setup of the monitoring system was hammered into the ground 2–5 m from the monitoring system. The time-lapse camera was mounted to the top of the angle bar and focused on the monitoring system. The cameras were programmed to take a picture every 15 min. This interval was chosen based on the data processing time for the photos, and on the power consumption of the cameras (with a 15-minute interval, the cameras run for about two months).

## 4. Evaluation of the Multi-Sensor Monitoring System 

### 4.1. Microcontroller Board and Data Logger Shield Combination 

The performance of the microcontroller board and data logger combination was evaluated in terms of its reliability to log the time and sensor data, and the accuracy of the logged time. The latter was expressed as the range and average clock drift (in minutes per month) of the RTCs.

The microcontroller board and data logger combination was able to log the time and data 98.1% of the time during the 2016 field season and 100% of the time during the 2017 field season. The clock drift of the RTCs ranged from 0.5 to 2.5 min per month (average of 1.3 min per month) for the 2016 field season and 0.5 to 3 min per month (average of 1.5 min per month) for the 2017 field season.

### 4.2. Sensors 

**Figure 12:**
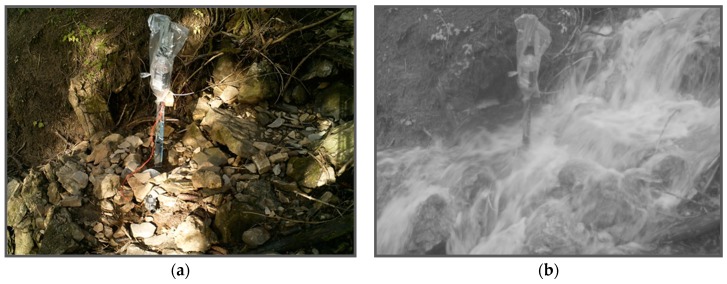
Two examples of time-lapse photos of one of the multi-sensor monitoring systems (2016 field season): (**a**) dry channel (state data: no water and no flow) and (**b**) flowing water (state data: water and flow).

The performance of the sensors was evaluated by comparing the state data derived from the sensor data to the state data derived from the photos taken by the time-lapse cameras (Figure 12). The state data from the photos was derived by manually scanning through the photos and noting the times of the state changes. The sensor performance was expressed as the percentage correct state data (i.e., the percentage of the state data derived from the sensor data that corresponded to the state data derived from the time-lapse photos) and the percentage correctly timed state changes (i.e. the percentage of the state changes derived from the sensor data that corresponded in timing with the state changes derived from the time-lapse photos). Furthermore, the sensors were evaluated on the type of errors they committed, by subdividing the errors into false positive errors (incorrect water or flow states in the state data derived from the sensor data) and false negative errors (incorrect no water or no flow states in the state data derived from the sensor data). The false positives and false negatives were expressed as the percentage of the total error count per sensor. 

For the 2016 field season, the percentage correct state data was higher than 90% for every sensor except for the float switch sensor (75.0%) (Table 2). The ER sensor performed best in this respect, with 99.9% correct state data. The performance of the sensors was poorer with respect to percentage correctly timed state changes, which was close to or less than 50% for all sensors, except for the ER sensor (93.5%). The temperature sensor performed poorest in this respect, with only 10.4% correctly timed state changes. The performance of the temperature sensor was poorest at locations with an episodic temporary stream regime. The performance of the flow sensor was poorest at locations that experienced low flows relatively often. For the other sensors, the level of performance was unrelated to their location in the catchment. 

Due to the modifications to the float switch sensor and the flow sensor in between the two field seasons, the performance of both sensors improved significantly for the 2017 field season. Comparable to the ER sensor, the percentage correct state data for these sensors was now almost 100% (Table 2). With respect to the percentage correctly timed state changes, the ER sensor and the flow sensor performed best (90.9% and 90.5% respectively). The change in the position of the temperature sensor after the first season improved the percentage correctly timed state changes to 23.6%, but overall the temperature sensor performed the poorest of all sensors during the 2017 field season, in particular at locations with an episodic temporary stream regime. For the other sensors, the level of performance was unrelated to the location in the catchment. 

The type of errors committed by the sensors were, for both field seasons, mostly false positives for the ER sensor, temperature sensor, and float switch sensor and solely false negatives for the flow sensor (Table 3).

## 5. Discussion

### 5.1. Microcontroller Board and Data Logger Shield Combination

The microcontroller board and data logger shield combination was chosen over the conventional, off-the-shelf data loggers that were used in previous temporary stream monitoring studies [49,50,61,62]. Unlike the conventional loggers, the microcontroller board and data logger shield combination can be custom programmed, which enables a wider range of data logging possibilities. While for this study the microcontroller board and data logger combination was programmed as an interval logger, for future studies it could also be programmed as a state or event logger (the interrupt pins on the microcontroller board can be used for state and event logging). For interval logging, custom programming offers infinite possibilities for the length of the logging interval. Furthermore, the interval can be programmed to be longer or shorter for specified times, (e.g., during base or storm flow) or to increase or decrease in length over time (e.g., during the rising and falling limbs of the hydrograph). For state logging, custom programming offers the possibility to assign custom state change thresholds, rather than having to work with pre-programmed thresholds (which is the case for most conventional loggers). Another advantage of the microcontroller board and data logger shield combination is the memory flexibility of the data logger shield. Conventional loggers often make use of built-in memory to store data, which is difficult to modify. The data logger shield, on the other hand, saves the data on an exchangeable SD-card. This allows the memory size to be adjusted based on the needs of the user. Finally, the microcontroller board and data logger shield combination is cheaper than commercial loggers. Not only is the combined price of a microcontroller board and data logger shield lower, but the microcontroller board also offers more connections for sensors, thus lowering the costs per sensor. The overall reduced costs per monitoring setup allows for higher spatial resolution monitoring.

The interval logging approach used in this study was chosen over a state logging approach as was used in several previous temporary stream monitoring studies [35,50,62]. The advantage of interval logging over state logging is that the first enables logging of raw sensor data. The availability of the raw sensor data allowed for data cleaning and defining catchment specific conversion filters prior to converting the data into state data. This improved the quality of the state data. In addition to that, the raw sensor data in combination with the state data helped to better assess the type of errors committed by the sensor. Bhamjee and Lindsay (2011) [35] argued that a state logging approach is preferable to an interval logging approach for temporary stream monitoring because the latter would quickly reduce memory capacity when measuring at short intervals. However, because the data logger shield allows the memory size to be adjusted, this was not an issue. 

The results of the field tests show that the microcontroller board and data logger shield combination was reliable, with close to no data logging failures. The 1.9% failed data logs for the 2016 field season were attributed to a single microcontroller board and data logger shield combination, which for unknown reasons stopped logging data for eight days in the middle of the field season and then continued to work again. There were no failures for the other microcontroller boards and data logger shield combinations. The RTC drift, on the other hand, was considerable. The average RTC drift for both field seasons was more than three times higher than the average clock drift measured for commercial pressure transducers in a study by Rau et al. (2019) [63]. Accumulating RTC drift over a relatively long period could be problematic when comparing the sensor data to data from other instruments with significantly smaller clock drifts. During the field tests, the RTCs were reset every month or two. For a period of this order, the amount of RTC drift was less than the logging interval time, which was considered acceptable. If, for a future project, regular resetting of the RTC is not an option, then it could be worth it to invest in a better RTC.

As the data storage setup allowed for years of storage, it was not required to go to the field frequently to collect the sensor data. However, to further simplify data collection and allow the ability to collect real-time data, the next step would be to add a module to the multi-sensor monitoring system that enables wireless data transmission. Such a module was not included in the current setup of the monitoring system because it would have significantly increased the power consumption and costs of the monitoring system. Furthermore, the limited reception in the study catchment would have been an issue for optimal data transfer. However, new developments in wireless technology will improve the power consumption, reception and costs of these modules, making it more practical and cost-effective to include them in future setups.

### 5.2. Sensors

The ER sensor performed well during both field seasons. The use of relatively long electrodes in combination with the catchment specific data filter, resulted in only a few errors. The few errors were related to instances where the data filter was not able to distinguish a damp sediment signal from a wet channel signal (false positives), and rainfall puddles for which the resistance was higher than the upper boundary set for wet channel conditions in the data filter (false negatives). To eliminate the first type of error, a housing could be added to the design. This would also simplify the data filter. However, with the current design and data filter, these errors were already sparse, and the small gain in performance will probably not outweigh the extra time and costs related to designing, creating, installing, and maintaining the housing.

The temperature sensor performed well with respect to the percentage correct state data but poor with respect to the percentage correctly timed state changes. The problems were similar to those encountered in previous studies [44,45]. Most errors were related to sudden weather-related changes in temperature and damp sediment on the sensors (false positives). The weather-related changes caused the state change timing in case of channel wetting to be too early. The damp sediment on the sensors caused the state change timing in case of channel drying to be too late. Both state change timing errors occurred equally frequent. In most cases, they were within two hours of the actual state change timing. This explains why even though the percentage correct state changes was low, the percentage correct state data was higher than 90% for both field seasons. Other errors were related to the minimum state duration settings of the data filter, which caused wet events shorter than 2.5 hours and dry events shorter than three hours to be omitted (false negatives and false positives, respectively). This is the reason for the particularly poor performance of the temperature sensor at locations with an episodic temporary stream regime. The change in position of the sensor after the first field season reduced the influence of damp sediment, which is reflected in the slightly improved percentage correctly timed state changes. The sensor performance can most likely be further improved by placing the temperature sensor in a housing to fully shield the sensor from sediment. Additionally, the parameters of the data filter could be improved. As the current parameters of the data filter were obtained by comparing the moving standard deviation temperature data with state data of the ER sensors for four monitoring locations, a comparison for more locations may yield better parameters. It remains, however, questionable if the performance of the temperature sensor can reach the same level as the ER and float switch sensor. On top of that, the conversion of the temperature data into state data is more subjective and time-consuming than for the other sensors and requires the state data of a separate sensor. 

The performance of the float switch sensor improved significantly after the modifications to sensor design in between the two field seasons. The replacement of the PVC pipe with the PLA housing, and the clip-on platform with the PLA platform, plus the addition of the filter sock to the setup, eliminated the housing and sediment related issues. Of the few errors for the 2017 field season, most were related to instances where water did not drain quickly enough from the housing and filter sock (false positives). This caused the state change timing in case of channel drying, to be too late. In a future setup, this could be improved by covering the housing with a filter sock with a slightly larger mesh. The other errors were attributed to a single float switch sensor, which in some instances switched at a water level higher than the 1 cm switch offset (false negatives). This caused the state change timing to be too late in case of channel wetting and too early in case of channel drying.

The performance of the flow sensor also improved significantly after the modifications to sensor design in between the two field seasons, specifically with respect to the percentage correctly timed state changes. The introduction of the PLA pipe fitting to the setup allowed the flow sensor to consistently detect low flows. The few errors for the 2017 field season were attributed to a single flow sensor, which in some instances did not record low flows (false negatives). This caused the state change timing to be too late when flow started and too early when flow ended. The errors of this single flow sensor were most likely related to a slight bend in the axis of the impeller, which could have made it harder for the impeller to spin properly.

When considering the performance of all the sensors, a combination of the ER and flow sensor would be optimal to provide information on the presence of water and the occurrence of flow. For future setups, the float switch sensor and temperature sensor could be excluded to save a bit more power during data logs. However, as the power draw and installation time is minimal for these sensors, they could be kept in the setup to provide backup state information, and temperature data that may be useful for other applications.

### 5.3. Power Efficiency 

The power-saving measures allowed the multi-sensor monitoring system to run for nine months on four lithium AA batteries at a five-minute logging interval. This level of power efficiency permits time and cost-effective, high spatiotemporal resolution monitoring. To further improve power efficiency, the voltage regulator of the microcontroller board, which is relatively inefficient at low current draws, could be replaced with a more efficient voltage regulator. 

Although the current setup is power-efficient, it could be considered to, in a future setup, power the multi-sensor monitoring system using a stand-alone power system consisting of rechargeable batteries in combination with small low-cost solar panels. Since the current draw of the monitoring system is very low, it would not be necessary for the batteries to charge fast. Therefore, this setup would most likely also work in forested settings, where the performance of solar panels is generally reduced. 

### 5.4. Field Setup 

The installation of the multi-sensor monitoring system took about two hours for one person. Most of this time was spent on the installation of the angle and crossbar, and the flow sensor. This installation time can be considered relatively long when many monitoring systems need to be installed; however, the setup proved to be very robust. The setup was able to withstand heavy rainfall, high flows, frozen streams, and a snowpack of up to a meter. The only observed damages to the system were a few small holes in parts of the tarp of the flow sensor that were not covered by the bed or bank material. These were most likely caused by small rodents, but did not influence the functioning of the system. Maintenance of the setup consisted mainly of removing sediment and organic debris from the mesh of the flow sensor. On some monitoring locations, this was needed after a large event or several medium events to prevent the mesh from getting clogged during the next event. While not problematic for this study catchment, this could be problematic when using the monitoring system in remote areas that cannot be accessed easily.

The robustness of the field setup in combination with the fact that the sensors (excluding the temperature sensor) performed well across the catchment during the 2017 field season, indicates that the multi-sensor monitoring system can be used in small temporary streams with a variety of stream regimes and characteristics. While the setup is most suitable for monitoring small temporary streams, it might also be possible to use the monitoring system in larger temporary streams with a stable thalweg and relatively low sediment load. 

### 5.5. Sensor Performance Evaluation Method

While using the time-lapse cameras to evaluate the sensor performance was time-consuming (installing the cameras and data processing of the photos), this method was preferred over a sensor-to-sensor comparison (paired sensor approach) as was used in the study by Bhamjee et al. (2016) [50]. Their approach expresses the sensor performance as the percentage of time that the combined output of two sensors, of which one can provide information on the presence of water and the other on the occurrence of flow, are valid or invalid. The combined output is considered valid for three combinations: no water and no flow, water and no flow, or water and flow, and invalid for one combination: no water and flow. However, this approach cannot account for sensor errors in the following situations:

1. Both sensors measure the incorrect state in case of a dry channel (combined sensor output: water and flow)
2. Both sensors measure the incorrect state in case of flowing water (combined sensor output: no water and no flow)
3. Only the flow sensor measures the incorrect state in case of flowing water (combined sensor output: water and no flow).

As the combined sensor output in these situations is valid according to the approach, the sensor performance will generally be overestimated. Using the time-lapse cameras provided a form of continuous direct observation that allowed to account for these errors.

The results of the error assessment underline the value of using time-lapse cameras for the evaluation of the performance of the sensors, over a sensor-to-sensor comparison. The ER sensor, temperature sensor, and float switch sensor mostly committed false positive errors and the flow sensor solely false negative errors. The combined output of these errors is water and no flow, which is a valid combined output according to the sensor-to-sensor comparison. Using this approach would therefore have resulted in an overestimation of the sensor performance.

While the time-lapse cameras allowed for a comprehensive evaluation of the sensor performance, a shorter time interval between the photos would have allowed for a more accurate evaluation of the performance of the sensors with respect to their ability to correctly time state changes. This would, however, have significantly increased the data processing time for the photos, and the number of field visits to change the batteries of the cameras. To reduce the data processing time of photos in future evaluation approaches with time-lapse cameras, a pattern recognition algorithm could be applied to the photos instead of scanning through the photos manually.

## 6. Conclusions

This study shows that the multi-sensor monitoring system, consisting of open-source and inexpensive technology, can be used to collect high spatiotemporal resolution information on the presence of water and the occurrence of flow in small temporary streams in mountainous headwater catchments. The microcontroller board and data logger shield combination was able to reliably log time and data and allows for more custom programmable data logging, more memory flexibility, and more sensors per logger than conventional loggers. The ER sensor and flow sensor performed best during the field tests and a setup with these sensors would suffice to monitor the three main hydrological states of temporary streams. The system was power efficient and the field setup robust. The time-lapse cameras were very valuable for the evaluation of the sensor performance, as a sensor-to-sensor comparison would have overestimated the performance of the sensors. Future improvements to the system would be the addition of a module that enables wireless data transfer, and possibly a better RTC to eliminate the necessity for regular clock resets. It is expected that the use of the multi-sensor monitoring system will aid to improve our understanding of the hydrological functioning of temporary streams.

## Figures and Tables

**Table 1:** Overview of the power saving measures and corresponding reductions in current draw during and in between data logs.

Setup	Current Draw During Data Logs (mA)	Current Draw in between Data Logs (mA)
Initial setup ^1^	55.3	53.3
1. Using Arduino Pro Mini	22.3 (−33.0)	20.3 (−33.0)
2. Powering down on-board peripherals	22.3	5.5 (−14.8)
3. Powering down sensors	22.3	2.1 (−3.4)
4. Removing power LEDs	20.4 (−1.9)	0.2 (−1.9)

^1^ Initial setup consisted of an Arduino Uno microcontroller board, the SD-card data logger shield, ER sensor, temperature sensor, float switch sensor and flow sensor.

**Table 2:** Sensor performance for the 2016 and 2017 field seasons.

	2016		2017	
Sensors	Correct State Data ^1^ (%)	Correctly Timed State Changes ^2^ (%)	Correct State Data ^1^ (%)	Correctly Timed State Changes ^2^ (%)
ER	99.9 (99.9–100)	93.8	99.9 (97.7–100)	90.9
Temperature	93.5 (84.6–100)	10.4	91.0 (40.3–100)	23.6
Float switch	75.0 (10.4–100)	25.0	99.8 (99.5–100)	84.9
Flow	94.2 (0.0–100)	56.1	99.9 (98.8–100)	90.5

^1^ The values in front of the brackets represent the percentage correct state data per sensor for all monitoring locations combined. The values between brackets represent the range of percentage correct state data per sensor for all monitoring locations. ^2^ The values represent the percentage correctly timed state changes per sensor for all monitoring locations combined. The total number of water/no water state changes was 48 in 2016 and 66 in 2017. The total number of flow/no-flow state changes was 41 in 2016 and 42 in 2017. Ranges for the percentage correctly timed state changes are not given because for some locations there were too few state changes for the percentage to be meaningful.

**Table 3:** Type of errors committed by the sensors for the 2016 and 2017 field seasons.

	2016		2017	
Sensors	False Positives ^1^ (%)	False Negatives ^1^ (%)	False Positives ^1^ (%)	False Negatives ^1^ (%)
ER	66.7	33.3	85.7	14.3
Temperature	91.8	8.2	88.7	11.3
Float switch	99.7	0.3	84.6	15.4
Flow	0.0	100	0.0	100

^1^ The values represent the percentage false positive and false negative errors of the total error count per sensor for all monitoring locations combined.

## Supplementary Materials

The following are available online at https://www.mdpi.com/1424-8220/19/21/4645/s1: Description S1: General description of the initial lab tests; Table S1: Descriptions of the low-cost sensors that were evaluated in the initial lab tests; Table S2: Performance results of the low-cost sensors that were evaluated in the initial lab tests; 3D-print object S1: Housing for the float switch sensor; 3D- print object S2: Platform for the float switch sensor; 3D-print object S3: Pipe fitting for the flow sensor; Arduino sketch S1: Operating program for the multi-sensor monitoring system.

## Appendix A

Circuit reference designator list:

BT	=	Battery
J	=	Connector
Q	=	Transistor
R	=	Resistor
RT	=	Thermistor
S	=	Switch
VR	=	Variable resistor
U	=	Integrated circuit
