# Supplementary material for: A Low-Cost, Multi-Sensor System to Monitor Temporary Stream Dynamics in Mountainous Headwater Catchments
Source: Sensors (Basel). 2019 Oct 25;19(21):4645. doi: 10.3390/s19214645 (PMC6864461; doi:10.3390/s19214645)
Supplement: Supplementary file 1 [file sensors-19-04645-s001.zip › Supplementary_Material_Manuscript_RSAssendelft_HJvanMeerveld_Sensors/Instructions_Supplementary_Materials.pdf]

Instructions for the Supplementary Materials

1. The STL files containing the 3D-print objects can be opened by any STL editor software.
2. The INO file containing the Arduino sketch can be opened using the open source Arduino Integrated Development Environment (IDE) software (version 1.8.9).
