# Supplementary material for: A Low-Cost, Multi-Sensor System to Monitor Temporary Stream Dynamics in Mountainous Headwater Catchments
Source: Sensors (Basel). 2019 Oct 25;19(21):4645. doi: 10.3390/s19214645 (PMC6864461; doi:10.3390/s19214645)
Supplement: Supplementary file 1 [file sensors-19-04645-s001.zip › Supplementary_Material_Manuscript_RSAssendelft_HJvanMeerveld_Sensors/Supplementary_Material_Description_S1_Tables_S1_and_S2.docx]

**Supplementary material**

**Description S1**

Nine low-cost sensors that had the potential to provide either information on the presence of water or the occurrence of flow were evaluated during initial lab tests (Table S1). The sensors were evaluated in terms of sensitivity and robustness (Table S2) by exposing them to alternating water/no water and flow/no flow conditions, and the influence of sediment, over a period of two weeks. Water/no water conditions were created by manually filling and draining a tank in which the sensors were placed, while flow/no flow conditions were simulated in a flume. The influence of sediment was tested by introducing sand and pebbles in the tank and flume. Ultimately, the ER sensor, temperature sensor, float switch sensor and flow sensor were considered suitable for further testing in the field (the last two after modifications to the original design). The other sensors were either not sensitive enough or not robust enough for long-term placement in streams.

**Table S1.** Descriptions of the low-cost sensors that were evaluated in the initial lab tests.

| Sensor | Design | State information | Principle |
| --- | --- | --- | --- |
| Temperature¹ | Epoxy-coated thermistor (temperature resistor) | Water/no water | The sensor measures the resistance of the thermistor, which is converted into temperature. The diurnal temperature signal of water is dampened in comparison to the temperature signal of air. |
| Water² | Module with interlaced ground and sensor cupper traces | Water/no water | The sensor measures the resistance between the traces. The presence of water reduces the resistance between the traces. |
| Moisture² | Two legged fork, which is laced with copper | Water/no water | The sensor measures the resistance between the legs. The presence of water reduces the resistance between the legs. |
| Electrical resistance³ | Two copper wires, which serve as electrodes | Water/no water | The sensor measures the resistance between the electrodes. The presence of water reduces the resistance between the electrodes. |
| Float switch^4^ | Float with integrated magnet, which slides over a vertical stem with reed switch circuit | Water/no water | The sensor measures if the reed switch circuit is open or closed. A rising water level moves the float up the vertical stem, causing the magnet to align with the reed switch and the circuit to open. |
| Liquid level¹ | Measurement tape with pressure sensitive resistor envelope | Water/no water | The sensor measures the resistance of the pressure sensitive resistor. Water pressure is higher compared to air pressure. The measured resistance can be converted into water level. |
| Ultrasonic^1^ | Module with ultrasonic transmitter, receiver and control circuit | Water/no water | The sensor measures the distance to a surface directly in front of it, such as the distance to the water surface in a stream or to the dry streambed. |
| Flow switch² | Pipe with magnetic valve and reed switch | Flow/no flow | The sensor measures if the reed switch circuit is open or closed. Flowing water forces the valve down the pipe, causing the magnet to align with the reed switch and the circuit to open. |
| Flow^5^ | Valve body, impeller with integrated ring magnet and Hall-effect sensor | Flow/no flow | The sensor measures electrical pulses. Flowing water spins the impeller and the magnet past the Hall-effect sensor, causing it to switch between ON and OFF states. These pulses can be counted over a specified time interval and converted into discharge. |

¹ Adafruit Industries, New York City, NY, USA

² SEEED Studio, San Leandro, CA, USA

³ Own design

⁴ Hamlin Electronics L.P., Lake Mills, WI, USA

^5^ YIFA Plastic Products Co., ltd, Yuè, Foshan, China

**Table S2**. Performance results of the low-cost sensors that were evaluated in the initial lab tests

| Sensor | Sensitivity | | Robustness | | Issues |
| --- | --- | --- | --- | --- | --- |
|  | NS | S | NS | S |  |
| Temperature | + | + | + | + | None. |
| Water | + | + | - | - | After several days in contact with water, the copper sensor traces started to oxidize and the sensor stopped working. |
| Moisture | + | + | - | - | After several days in contact with water, the copper lacing on the legs started to oxidize and the sensor stopped working. |
| Electrical resistance | + | + | + | + | None. |
| Float switch | + | - | + | + | Sediment prevented the float from moving up and down the vertical stem. |
| Liquid level | - | - | + | + | The sensor did not register water levels below 4 cm. |
| Ultrasound | + | - | - | - | Sediment accumulation resulted in false water readings during no-water conditions. The sensor was not resistant to moisture. |
| Flow switch | - | - | + | + | A relatively high discharge was required to move the magnetic valve. Coarse sediment particles got stuck in the pipe and blocked the valve. |
| Flow | + | - | + | + | Coarse sediment particles got stuck in the valve body and blocked the impeller. |

NS = in non-sediment conditions

S = in sediment conditions

+ = sensor performs well

- = sensor performs poorly
